# Concurrent Rabies and Canine Distemper Outbreaks and Infection in Endangered Ethiopian Wolves

**DOI:** 10.3201/eid3012.240432

**Published:** 2024-12

**Authors:** Jorgelina Marino, Elizabeth F.R. Preston, Muktar Abute, Alo Hussein, Fekede Regassa, Asefa Deressa, Eric Bedin, Ashley C. Banyard, Anthony R. Fooks, Claudio Sillero-Zubiri

**Affiliations:** University of Oxford and Ethiopian Wolf Conservation Programme, Tubney, UK (J. Marino, E.F.R. Preston, C. Sillero-Zubiri); University of Oxford and Ethiopian Wolf Conservation Programme, Robe-Bale, Ethiopia (M. Abute, A. Hussein, E. Bedin); Ethiopian Wildlife Conservation Authority, Addis Ababa, Ethiopia (F. Regassa); Ethiopian Public Health Institute, Addis Ababa (A. Deressa); Animal and Plant Health Agency, Weybridge, UK (A.C. Banyard, A.R. Fooks)

**Keywords:** rabies, canine distemper, Ethiopian wolf, viruses, outbreak, Ethiopia

## Abstract

Intensive disease surveillance in an endangered population of Ethiopian wolves provided evidence of concurrent outbreaks of rabies and canine distemper viruses in 2019, including co-infection in an individual animal. Disease surveillance and intensive monitoring of wolf packs in Ethiopia were essential in detecting the concurrent outbreaks and enabled accurate assessment of disease from both pathogens. The study highlights the risk posed to endangered populations that are susceptible to, or live in areas with, reservoir hosts for canine distemper and rabies viruses. Instances of concurrent distemper and rabies outbreaks appear unusual in the existing literature; modeling for one disease might underestimate the risk for extinction. Concurrent outbreaks may have a larger effect than single-disease outbreaks, even in a population that has partial vaccination coverage. Researchers studying wildlife populations from a conservation perspective should be aware that both diseases can strike at once where susceptible populations exist.

Infectious diseases are an increasing threat to wildlife, domestic species, and humans around the world (*1*,*2*). Although the recent SARS-CoV-2 pandemic has demonstrated the effects of zoonotic transmission of viral diseases from wildlife to the human population, the dynamics of pathogen transmission between domesticated animal populations and sylvatic populations can drive the emergence of infectious diseases in wildlife species, with potentially catastrophic outcomes (*2*). The risk from disease is greatest for small populations that live in close proximity to humans and their livestock and companion animals; small populations face a greater likelihood of stochastic population change caused by disease spillover that can further reduce population size or even lead to extinction (*1*,*2*). Domestic dogs are a particular danger to wild carnivores and are linked to many incidences of pathogen transmission that cause conservation issues (*3*–*7*), because increasing populations of free-ranging dogs in rural and wild areas lead to frequent spillovers of disease into wildlife (*3*,*4*).

Rabies affects many mammal species, including humans and livestock. When the virus persists within domestic dog populations, it is often a public health and conservation threat (*8*). Rabies virus is a particular concern for several threatened carnivores (*9*) including endangered African wild dogs (*Lycaon pictus*) (*10*–*12*) and Ethiopian wolves (*Canis simensis*) (*13*–*16*). Rabies virus is one of the few viral pathogens that almost always causes a fatal clinical disease from a productive infection. Other diseases of domestic and wild animals are emerging as threats to endangered species worldwide, including canine distemper virus (CDV). CDV is a highly infectious virus that causes a profound immunosuppression after infection, often leading to secondary opportunistic infections driving high rates of illness and death (*17*). In contrast to rabies virus, CDV can circulate in a mild state; disease outcomes are dependent on viral and host genetics, immunostatus, nutritional status, and species susceptibility (*18*). In recent years, spillovers of CDV from domestic dogs have been associated with severe declines in wild carnivore populations worldwide. Outbreaks have been recorded in African wild dogs (*5*,*6*,*19*,*20*), lions (*Panthera leo*) (*19*,*21*), black-footed ferrets (*Mustela nigripes*) (*22*), Ethiopian wolves (*16*,*23*), Santa Catalina island foxes (*Urocyon littoralis*) (*24*), Caspian seals (*Pusa caspica*) (*25*), spotted hyenas (*Crocuta crocuta*) (*26*), Amur tigers (*Panthera tigris altaica*) (*27*), and gray wolves (*Canis lupus*) (*28*).

Across rural Africa, the interface between domestic and wild animals provides many opportunities for transmission of infectious viral diseases; Ethiopia experiences some of the highest incidences of rabies in the world (*29*). In this context, there is an unexplored risk for population decline or extinction from concurrent disease outbreaks in wildlife. Several case studies have investigated the effect of disease on population extinction risk and management strategies to reduce this (*30*–*32*), but they tend to focus on a single disease in the system or the presence of a single disease at a time. Diseases, however, can function differently in the same population; for example, CDV and rabies differ in their reservoir species and dynamics in African wild dogs (*6*,*7*). Carnivore species have experienced outbreaks of each of these diseases on separate occasions or in different populations (*5*,*6*,*10*–*12*,*16*,*19*–*21*,*23*), yet concurrent infections have only been recorded in northern raccoons (*Procyon lotor*), red foxes (*Vulpes vulpes*), and striped skunks (*Mephitis mephitis*), in the United States (*33*–*35*). There is a critical knowledge gap on the demographic outcomes of multiple viral infections in wildlife, in the face of increasing frequency of CDV and resulting illness in wild carnivores (*36*,*37*). Studies that report co-infection rarely address demographic outcomes and are often limited to either individual deaths (*38*) or report co-infections not linked to disease outbreaks (*39*).

The risk for concomitant outbreaks should be considered and explored further in vulnerable populations, particularly those of conservation concern (*16*,*40*). Concurrent outbreaks, like single outbreaks, can easily be unnoticed unless disease monitoring and diagnoses are sustained throughout an outbreak, and testing is performed for multiple pathogens. Here, we report concurrent outbreaks of rabies and distemper among Ethiopian wolves, describing the spatial and temporal spread of deaths and the overall impacts on the host population. Individually, those diseases have had a substantial impact upon the Ethiopian wolf population in the Bale Mountains of Ethiopia (the largest population, with more than half the surviving 500 wolves), and consecutive outbreaks have led to extinction of one of the smallest populations (*16*). 

## Materials and Methods

### Wildlife Population

Monitoring of the Ethiopian wolves in the Bale Mountains has been continuous since 1997, with a long-term focus on 2 core subpopulations, Sanetti Plateau and Web Valley; the neighboring subpopulations of Morebawa (connected to the Web Valley by the Genale Corridor) and Chafadalacha were also monitored. Subpopulations are groups of neighboring wolf packs separated from others by geographic barriers or habitat bottlenecks. A team of 6–8 experienced monitors routinely observe Ethiopian wolf packs on foot or horseback, during the daytime, following standard protocols (*15*,*41*). Disease surveillance is also part of the routine field monitoring; carcasses are found opportunistically or in response to reports by park rangers and members of the local community, and searches intensify afterward. Well-established disease alert networks, involving local communities, animal health offices and park staff, also contribute to detection of diseases among local dog populations. We implemented oral rabies vaccination campaigns in 2018 and 2019, concentrated in Morebawa (10 packs) and the Web Valley (2 packs); no packs in the Sanetti Plateau were vaccinated before the outbreak (Ethiopian Wolf Conservation Programme, unpub. data). A limited trial of a CDV vaccine targeting packs in Chafadalacha (2 packs) and Morebawa (1 pack) was also performed.

### Observations and Data Collection

In March 2019, an Ethiopian wolf carcass was found in the Bale Mountains, and an infectious disease was suspected as the cause of death; subsequently, monitoring for carcasses increased across the area. We conducted detailed necropsy examinations of carcasses whenever possible and collected tissue samples, including lymph node, lungs, spleen, and brain. We targeted tissue collection to ensure that organs assessed for infection were appropriate for each pathogen tested. The estimated time of death was assigned into categories according to the level of decomposition: within 1 day, 1–2 days, within 1 week, within 1 month, and >1 month.

### Laboratory Procedures

We tested samples for both rabies and CDV viral nucleic acids because these diseases have previously been detected in the population and caused outbreaks of disease in this area. For evaluation of viral nucleic acid within samples, we extracted total RNA as described previously (*42*); all suspect material was handled within a Biosafety Level 3 facility at the Animal and Plant Health Agency (Weybridge, UK). To detect rabies virus nucleic acid, we used a pan-lyssavirus SYBR green reverse transcription PCR as described previously (*43*). We detected canine distemper virus using an in-house real-time reverse transcription PCR (*42*). 

#### Disease Mapping and Population Assessment

We mapped the spread of diseases across the Bale Mountains wolf population from the global positioning satellite locations of carcasses and adjusted by the estimated time of death. We derived complementary data on population change from intensive monitoring of packs in the 2 core areas, Web Valley and Sanetti Plateau, including the complete set of neighboring packs (8 in Web Valley and 5 in Sanetti Plateau). Those social groups and their territories were typically stable, which aided us in total counts; sightings of large and complete groups were common, particularly during early morning and evening communal greetings, during pack territorial patrols, and around dens during the breeding season. We followed standard protocols for close observations of focal packs throughout the year to obtain accurate information on their size, composition, and reproductive success; observation enabled us to perform complete enumerations of wolves into age and sex classes and to note individuals recognized by ear marks or morphologic clues (*41*). We assessed population change as a result of the outbreaks by comparing the total number of wolves and the number of wolves of different age and sex categories, compared before and after the outbreaks, based on the estimated time of death of the first and the last carcass found.

## Results

### Deaths

During March 2019–November 2019, we found 57 carcasses and observed another 5 wolves with advanced clinical signs consistent with an infectious agent (Table 1; Appendix Table 1). Wolves died in at least 19 packs in 4 subpopulations: Sanetti Plateau, Chafadalacha, Web Valley, and Morebawa. We extracted samples for laboratory analyses from 19 of those carcasses; other carcasses were in an advanced state of decomposition or severely scavenged. Seven animals tested positive for rabies virus and 13 tested positive for CDV, 1 individual tested positive for both pathogens. In the animals with confirmed diagnoses, death caused by distemper was higher than death apportioned to rabies. However, we were unable to extrapolate those findings to populations because less than half of the carcasses detected could be tested for diseases, but the number tested is still significant in comparison with data from other studies.

**Table 1:** Characteristics of Ethiopian wolf carcasses found in the Bale Mountains in study of concurrent outbreaks of rabies and canine distemper, Ethiopia, 2019*

Pack	Carcasses found	Known sex			Known age†			Carcasses tested	Rabies positive	CDV positive
		M	F		Adult	Subadult	Juvenile			
Sanetti Plateau subpopulation										
Badagasa	8	0	3		1	1	2	2	1	1‡
Batu	3	0	0		2	0	0	0	0	0
BBC	4		3		2	0	1	2	0	2
Bilisa	1	1	0		1	0	0	1	0	1
Buyamo	1	0	0		0	0	0	0	0	0
Garba Gurracha	1	0	0		0	1	0	0	0	0
Chafadalacha subpopulation										
Chafadalacha	1	0	0		0	0	0	0	0	0
Konteh	2	0	0		0	1	0	0	0	0
Web Valley subpopulation										
Alandu	1	0	1		0	0	1	1§	1§	1§
Bowman	4	2	1		1	1	2	2	1‡	1
Gata	1	1	0		0	1	0	0	0	0
Habale	1	0	0		1	0	0	0	0	0
Hangafo	2	1	1		1	0	1	1	1	0
Mckenna	1	0	0		1	0	0	0	0	0
Megity	10	5	1		3	2	1	4	1	3
Megity3	9	4	1		4	2	1	4	1	3
Tarura	2	2	0		2	0	0	1	1	0
Unknown	1	1	0		0	0	1	0	0	0
Genale Corridor subpopulation										
Genale	3	0	0		1	2	0	0	0	0
Morebawa subpopulation										
Gurati	1	0	1		0	1	0	1	1	1
Totals	57	17	12		20	12	10	19	7	13

*CDV, canine distemper virus, †Adult, >2 y; subadult, 1 to <2 y; juvenile, <1 y. ‡Indicates the first confirmed case of each disease. §Indicates case of co-infection.

### Patterns of Disease Spread

**Figure 1:**
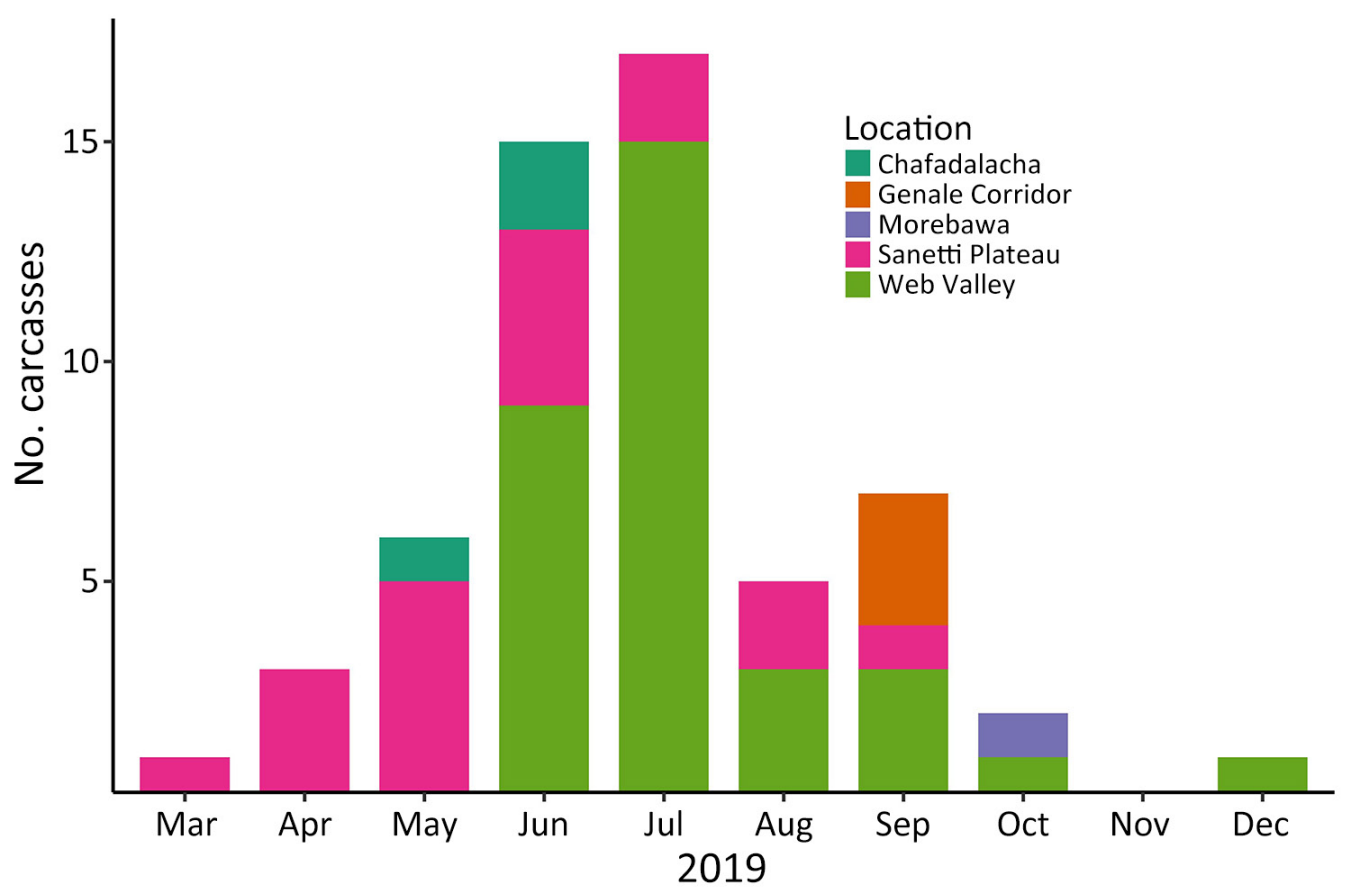
Carcasses of Ethiopian wolves retrieved in the Bale Mountains, by month and subpopulation, in a study of concurrent rabies and canine distemper outbreaks, Ethiopia, 2019. Estimated time of death determined from postmortem observations.

**Figure 2:**
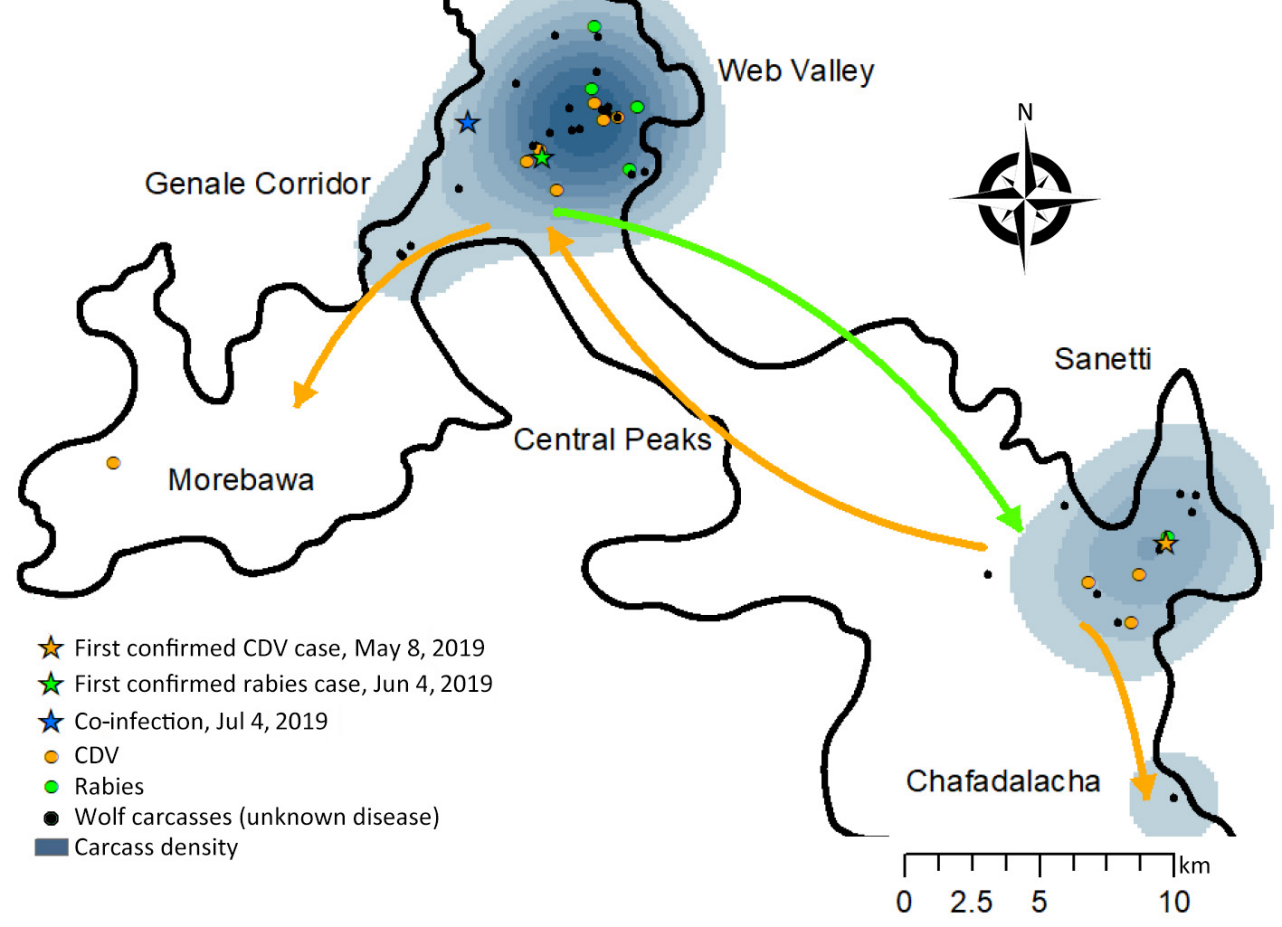
Location of Ethiopian wolf carcasses found in the Bale Mountains in a study of concurrent rabies and canine distemper outbreaks, Ethiopia, 2019. Shades of blue represent the kernel density distribution of all observed carcasses. Arrows indicate the direction of spread of infections as revealed by positive cases of each disease through time and across subpopulations. CDV, canine distemper virus.

**Figure 3:**
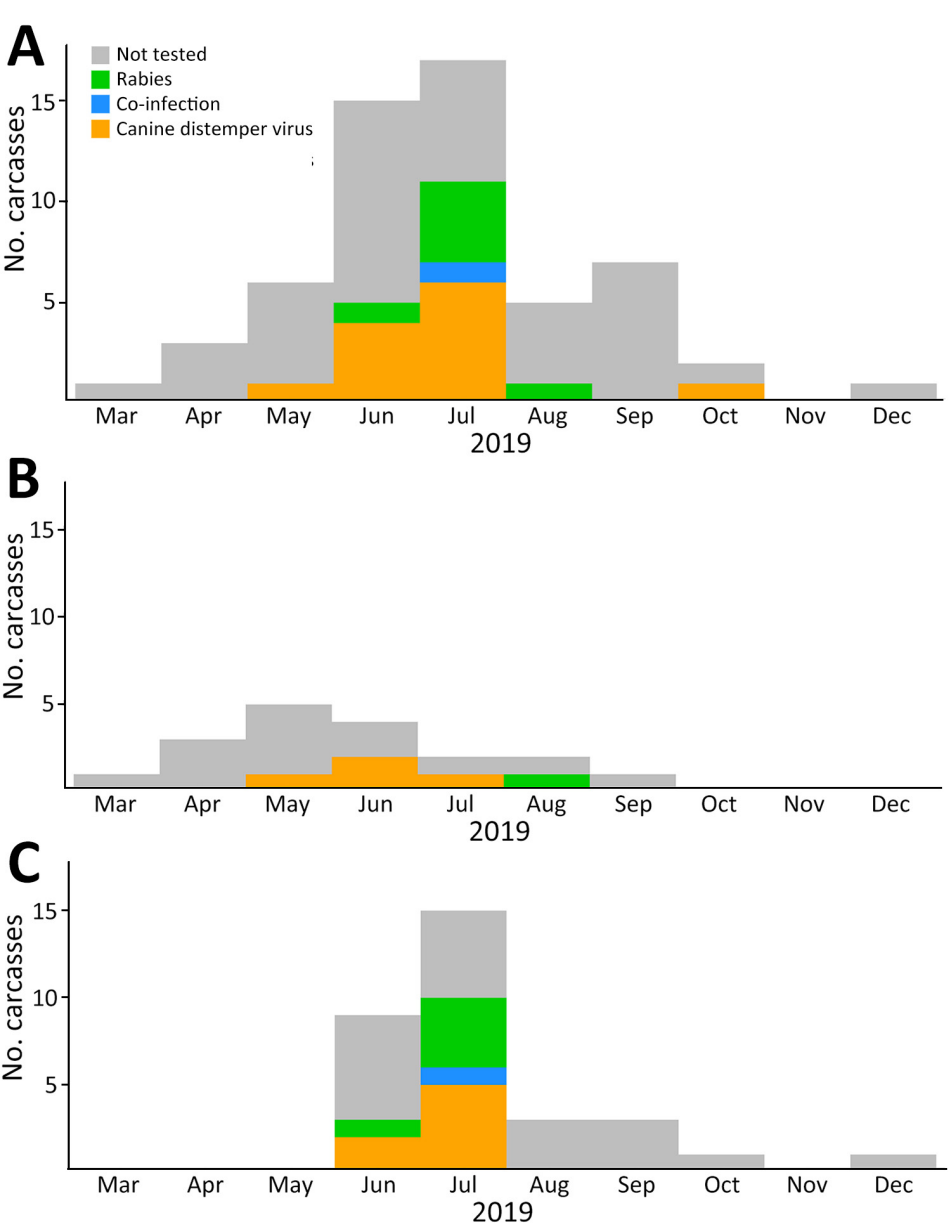
Number of carcasses retrieved per month in a study of concurrent rabies and canine distemper outbreaks in Ethiopian wolves, Ethiopia, 2019. Estimated time of death determined from postmortem observations. A) Full wolf population in the Bale Mountains. B) Sanetti Plateau subpopulation. C) Web Valley subpopulation.

The timing and location of carcasses encountered suggest pathways of disease dispersion across subpopulations (Figures 1–3). The center of infection was in the Sanetti Plateau, where the first 2 carcasses were found in March and April 2019, but CDV was first confirmed from a carcass sampled in May 2019. Overall mortality rate peaked in July 2019, five months after the onset of the outbreak, by which time disease had spread southwards to Chafadalacha and to the more distant Web Valley (Figures 2 and 3). At the peak of the outbreak, both CDV and rabies were common in Web Valley, whereas rabies virus was not detected in Sanetti Plateau until a month later (Figure 3). The spread of rabies and distemper appeared to occur in both directions, indicating different origins for the initial infections (Figure 2).

During August–October 2019, disease spread along the Genale habitat corridor connecting Web Valley with the Morabawa subpopulation; at least 1 death from distemper was confirmed toward the end of the outbreak (Figures 1, 2). Monitoring effort in Morabawa was relatively low in comparison with that in Web Valley and Sanetti Plateau. 

### Population Effects

The number of carcasses encountered (n = 57) provided an indirect and partial measure of overall mortality during the outbreaks (Table 1). We assessed local population effect from total counts of Ethiopian wolves in 2 core monitoring areas by comparing pack compositions before and after the outbreaks in the Sanetti Plateau (5 packs) and Web Valley (8 packs) subpopulations (Table 2). We noted a total of 64 animals unaccounted for in the 2 subpopulations, where 50 carcasses had been found: 17 unaccounted for in the Sanetti Plateau, representing 60% decline, and 47 unaccounted for in the Web Valley, representing 53% decline. 

**Table 2:** Changes in composition of wolf packs in Ethiopian wolves before and after concurrent outbreaks of rabies and canine distemper, Ethiopia, 2019*

Pack	Before outbreaks								After outbreaks			
	Adult			Subadult		Juveniles	Sex ratio		Adult/subadult		Subadults†	Sex ratio
	M	F		M	F				M	F		
Sanetti Plateau subpopulation												
Bagadasa	3	2		2	2	4	1.25		1	1	1	1
Garba Gurracha	2	1		0	1	0	1		2	1	0	2
Batu	2	2		1	1	0	1		2	1	0	2
BBC	3	2		1	2	0	1		2	1	0	2
BBC2	3	2		0	0	5	1.5		1	1	1	1
Total	22			10		9			13		2	
Web Valley subpopulation												
Alando	2	2		0	0	4	1		2	1	1	2
Bowman	4	7		2	2	5	0.666667		1	1	0	1
Habale	4	3		2	2	4	1.2		2	1	2	2
Hangafo	2	1		2	2	3	1.333333		2	1	0	2
Mckenna	3	2		1	1	0	1.333333		2	1	0	2
Megity	3	3		2	2	3	1		2	1	0	2
Megity 3	3	2		2	2	5	1.25		0	0	0	
Tarura	6	5		2	2	5	1.142857		3	4	4	0.75
Total	52			26		29			24		7	

*Sex ratio calculated as the ratio between male and female wolves >1 y of age. It excludes juveniles at the time of the outbreaks because they are difficult to sex when young, †These subadults were juveniles before the outbreak.

We assessed mortality patterns in 29 carcasses that could be classified by age and 42 by sex (Table 1). More carcasses were from adult wolves (>2 years of age), followed by subadults (1–2 years of age) and then juveniles (<1 year of age) (Table 1), and appeared to be split unevenly between the sexes (17 male animals, 11 female, and 29 unknown). Of the 13 carcasses tested positively for CDV, 6 were adults, 4 subadults, and 3 juveniles; 6 were female and 7 male). We confirmed rabies in samples from 3 adults and 4 juveniles but no subadults, albeit on a small sample (n = 7). The animal that tested positive for both diseases was a juvenile female.

Mortality inferred by changes in pack composition (Table 2) was consistent with the age distribution of carcasses: of the wolves missing in the population, 54 were adults, 40 subadults and 16 juveniles. In the Web Valley subpopulation, death rates were 53% among adults and 73% among subadults. In the Sanetti Plateau subpopulation, death rates were 41% among adults and 80% among subadults. More female wolves were missing than males (38 females vs. 35 males). 

## Discussion

Intensive monitoring of an Ethiopian wolf population provided confirmation of concurrent outbreaks and co-infection with rabies virus and CDV in an endangered mammalian species, and new information on population-level impact. Our findings support expectations that concurrent outbreaks can be more damaging to the survival of the Ethiopian wolf than single disease outbreaks (*16*,*40*); that expectation may also apply to other species that can contract both of these diseases, including African wild dogs, lions, and other carnivores. Studies of viral infections in wildlife are rare for several reasons, and therefore the knowledge gleaned from the concurrent outbreaks has important implications for disease surveillance and control, as well as some limitations.

Spreading monitoring effort across the extent of a population and over time enabled detection of multiple pathogens which could otherwise be missed, and we found only 1 other incidence of co-infection in an individual *33*). Indeed, because rabies and CDV are not uncommon diseases among wild carnivores and affect many of the same species, a likely reason that concurrent outbreaks are rarely reported is that they go unmonitored, which can happen if monitoring is concentrated in 1 subpopulation and misses 1 disease initially, or if all deaths are assumed to be attributable to a single disease. In our study, sustained and intensive monitoring maximized detection of deaths across the landscape, enabling us to recover 57 wolf carcasses; we found no evidence of deaths in other species. More often, most carcasses go undetected or the identification of the sex or age of a carcasses is impaired because of late detection. Time periods leading to death from rabies virus infection for canine species is typically within 2 weeks of infection. In contrast, CDV infection can cause clinical disease within a few days of infection and either results in clearance and survival from infection or progresses to a severe disease characterized by an acute leukopenia that may lead to secondary infections and death. Timelines for infection for CDV can vary, but in cases in which the disease leads to death, the timeline will often be in a similar time period to that observed with rabies virus infection. Carcass detection is inherently imperfect and depends on the monitoring effort.

The risks posed by concomitant outbreaks include wolf deaths lasting over a longer period and spreading further geographically than previously known. The carcasses detected in the Bale Mountains showed that this combined outbreak lasted >220 days and affected 4 subpopulations, compared with previous records of single-pathogen outbreaks among Ethiopian wolves shown to last for 3 months (*14*,*15*,*42*,*44*) and affecting 1 (*44*), 2 (*14*,*15*,*42*) or 3 (*42*) subpopulations. The unprecedented duration of the event was likely because of the overlapping outbreaks starting at slightly different times, as indicated by the timing and location of carcasses and adjusted by estimations of time since death from postmortems or body remains. The reconstructed pathways of disease spread indicated 2 different geographic origins and directions of spread for the rabies and CDV outbreak. Those chains of transmission between subpopulations coincide with those observed in previous outbreaks, involving an initially quick transmission within packs because of social bonds and shared space within pack territories, followed by transmission to neighboring packs. The absence of observations or reports of CDV or rabies cases among domestic dogs or other carnivore species living within or in close vicinity of Ethiopia wolf territories supports the wolf-to-wolf transmission pathway and coincides with descriptions of previous outbreaks. Although whole-genome sequencing and phylogenetic analysis will ultimately infer transmission dynamics, our findings were supported by a rate of carcass detection and diagnoses that we had not previously found in any wild population affected by these viruses.

Despite the large number of Ethiopian wolf deaths and disappearances during the concurrent outbreaks, declines of 53% and 60% in the 2 core subpopulations were comparable to death rates recorded during previous CDV outbreaks (43%–68% decline) (*42*) and slightly lower than most rabies outbreaks (49%–77% decline) (*14*,*15*,*44*). The implication that a combined outbreak might not be necessarily worse than a single-disease outbreak, however, is misleading; a degree of herd immunity existed in the population at the time of the outbreaks. Individually identifiable wolf carcasses that were tested for either disease (n = 6) had not been vaccinated against the disease for which they tested positive but had often been vaccinated against the other disease. More in-depth analyses would be needed to ascertain the effect of the previous vaccinations in the study population. Whereas rabies often causes overt clinical disease with an inherent zoonotic risk, the dynamics of CDV can be difficult to understand without serosurveillance because the virus can circulate in wild populations subclinically.

Population declines of the severity reported here highlight concern for any threatened species that exists in small populations such as the Ethiopian wolf. In the past, consecutive outbreaks of distemper and rabies led to the smallest population of this species becoming functionally extinct in Delanta (*16*). Ongoing oral vaccination of Ethiopian wolves against rabies have the potential to reduce risks of populations going extinct; it is a crucial intervention where rabies remains endemic in terrestrial carnivores in vast areas (*45*). Approaches to control rabies are relatively advanced and accessible because of the virus’s relevance to human and livestock health (*46*). Tools to control distemper in wildlife are less developed, because it generally does not infect humans and is therefore considered to be of lesser importance. Canine distemper vaccines have been available for decades but are rarely used in wildlife, and their efficacy is not well known, in part due to the complexity of CDV dynamics in natural ecosystems (*18*). A CDV vaccine is currently under trial (Ethiopian Wolf Conservation Programme, unpub. data).

Knowledge of the age and sex composition in disease-related deaths can also help deduce disease dynamics and immunity (*47*) to investigate relative risks and fitness costs of infection in social species (*26*). By combining information from carcasses with our knowledge of Ethiopian wolf packs’ demography, this study provides insights into the effects of CDV in wildlife populations, a neglected area of research. Statistical analyses by the age and sex of carcasses would be questionable because a large proportion of the deceased animals were of unknown age or sex; however, assessing such data is valuable because we know of no comparable level of disease surveillance and carcass recovery in other wildlife populations. Positivity for CDV spanned across age classes and was highest among adults, in itself not an indication of age-biased transmission or mortality because adults make a large proportion of the Ethiopian wolf population (*48*). When compared with single CDV outbreaks in Ethiopian wolves, these concurrent outbreaks were still unusual in the proportion of adult deaths recorded (47% adult mortality averaged across 2 subpopulations, compared with 34% during a CDV outbreak in 2006 and 39% in 2010). Subadult mortality rate was comparatively lower, 77% average, compared with 83% in the 2006 CDV outbreak and 87% in 2010. Although fatalities could not always be assigned to infections by rabies virus or CDV, high adult mortality is ultimately expected to reduce the capacity for recovery of this population (*48*) because it is more likely to lead to the extinction of a pack (*48*). The mortality rate among female wolves is higher than expected, given that there are more males in the populations we studied, which can also slow down population growth by limiting the effective population size.

In conclusion, this study highlights the risk to susceptible populations of threatened carnivores from concurrent infection from multiple pathogens. CDV and rabies viruses can strike at the same time and have a large effect even in a population that has partial vaccination coverage. Vaccination strategies using multivalent rabies/CDV vaccines should be favored when possible, in light of the possibility of concurrent outbreaks; the simultaneous impact of both diseases should be included in future models of vaccination programs and extinction risk of susceptible endangered populations. Existing models applied to Ethiopian wolf populations should be updated to integrate more and new information on potential multiple hosts of CDV, acquired immunity (*49*) and effectiveness of ongoing oral rabies vaccinations (*50*). The case of the Ethiopian wolf highlights the need for effective monitoring of endangered wildlife populations at risk for co-infections so that concurrent outbreaks are detected and their true impacts assessed accurately and incorporated into models to avoid underestimating the risk for extinction from disease.

Appendix Additional information about concurrent rabies and canine distemper outbreaks and infections in Ethiopian wolves, Ethiopia.
