# Supplementary material for: Concurrent Rabies and Canine Distemper Outbreaks and Infection in Endangered Ethiopian Wolves
Source: Emerg Infect Dis. 2024 Dec;30(12):2567–5276. doi: 10.3201/eid3012.240432 (PMC11616644; doi:10.3201/eid3012.240432)
Supplement: Appendix — Additional information about concurrent rabies and canine distemper outbreaks and infections in Ethiopian wolves, Ethiopia. [file 24-0432-Techapp-s1.pdf]

Article DOI: https://doi.org/10.3201/eid3012.240432

EID cannot ensure accessibility for supplementary materials supplied by authors. Readers who have difficulty accessing supplementary content should contact the authors for assistance.

Concurrent Rabies and Canine Distemper Outbreaks and Infection in Endangered Ethiopian Wolves

Appendix

Appendix Table. Ethiopian wolf carcasses found in the Bale Mountains*

Date found	Subpopulation	Pack	Age	Sex	Tested for rabies	Tested for CDV
28-Mar-19	Sanetti Plateau	BBC	A	F	Not tested	Not tested
29-Apr-19	Sanetti Plateau	BBC	U	U	Not tested	Not tested
11-May-19	Sanetti Plateau	Badagasa	S	F	Negative	**Positive**
23-May-19	Sanetti Plateau	Badagasa	U	U	Not tested	Not tested
24-May-19	Sanetti Plateau	Badagasa	A	U	Not tested	Not tested
31-May-19	Sanetti Plateau	Badagasa	U	U	Not tested	Not tested
1-Jun-19	Sanetti Plateau	Badagasa	U	U	Not tested	Not tested
11-Jun-19	Web Valley	Megity	A	F	Negative	**Positive**
11-Jun-19	Web Valley	Bowman	J	M	**Positive**	Negative
20-Jun-19	Sanetti Plateau	Bilisa	A	M	Negative	**Positive**
21-Jun-19	Chafadalacha	Chafadalacha	U	U	Not tested	Not tested
23-Jun-19	Sanetti Plateau	Badagasa	U	U	Not tested	Not tested
26-Jun-19	Sanetti Plateau	Batu	U	U	Not tested	Not tested
27-Jun-19	Sanetti Plateau	BBC	A	F	Negative	**Positive**
29-Jun-19	Web Valley	Bowman	A	F	Not tested	Not tested
29-Jun-19	Web Valley	Megity	A	M	Negative	**Positive**
9-Jul-19	Web Valley	Megity	S	M	Negative	**Positive**
11-Jul-19	Web Valley	Habale	A	U	Not tested	Not tested
11-Jul-19	Web Valley	Alandu	J	F	**Positive**	**Positive**
20-Jul-19	Web Valley	Megity3	J	U	Not tested	Not tested
20-Jul-19	Web Valley	Megity3	A	F	**Positive**	Negative
20-Jul-19	Web Valley	Unknown	J	M	Not tested	Not tested
20-Jul-19	Web Valley	Megity3	U	U	Not tested	Not tested
21-Jul-19	Web Valley	Megity3	A	M	Negative	**Positive**
21-Jul-19	Web Valley	Bowman	J	M	Negative	**Positive**
21-Jul-19	Web Valley	Megity3	A	M	Negative	**Positive**
21-Jul-19	Web Valley	Megity3	S	M	Negative	**Positive**
21-Jul-19	Sanetti Plateau	BBC	J	F	Negative	**Positive**
21-Jul-19	Web Valley	Megity 3	A	U	Not tested	Not tested
21-Jul-19	Web Valley	Megity 3	S	M	Not tested	Not tested
22-Jul-19	Web Valley	Gata	S	M	Not tested	Not tested
22-Jul-19	Chafadalacha	Konte	S	U	Not tested	Not tested
24-Jul-19	Chafadalacha	Konte	U	U	Not tested	Not tested
23-Jul-19	Web Valley	Makenna	A	U	Not tested	Not tested
23-Jul-19	Web Valley	Hangafo	J	F	**Positive**	Negative
25-Jul-19	Sanetti Plateau	Batu	A	U	Not tested	Not tested
24-Jul-19	Sanetti Plateau	Garba Gurracha	S	U	Not tested	Not tested
29-Jul-19	Web Valley	Megity	A	M	**Positive**	Negative
4-Aug-19	Web Valley	Hangafo	A	M	Not tested	Not tested
7-Aug-19	Web Valley	Tarura	A	M	**Positive**	Negative
19-Aug-19	Web Valley	Megity	J	M	Not tested	Not tested
19-Aug-19	Web Valley	Tarura	A	M	Not tested	Not tested

Date found	Subpopulation	Pack	Age	Sex	Tested for rabies	Tested for CDV
26-Aug-19	Sanetti Plateau	Badagasa	J	F	**Positive**	Negative
17-Aug-19	Sanetti Plateau	Badagasa	J	F	Not tested	Not tested
1-Sep-19	Sanetti Plateau	Buyamo	U	U	Not tested	Not tested
1-Sep-19	Web Valley	Megity	U	U	Not tested	Not tested
17-Sep-19	Web Valley	Megity	S	M	Not tested	Not tested
21-Sep-19	Sanetti Plateau	Batu	A	U	Not tested	Not tested
25-Sep-19	Web Valley	Megity3	U	U	Not tested	Not tested
8-Oct-19	Morebawa	Gurati	S	F	Negative	**Positive**
9-Oct-19	Genale Corridor	Genale	S	U	Not tested	Not tested
9-Oct-19	Genale Corridor	Genale	S	U	Not tested	Not tested
10-Oct-19	Genale Corridor	Genale	A	U	Not tested	Not tested
21-Oct-19	Web Valley	Megity	U	U	Not tested	Not tested
24-Oct-19	Web Valley	Megity	U	U	Not tested	Not tested
26-Nov-19	Web Valley	Megity	U	U	Not tested	Not tested
9-Jan-20	Web Valley	Bowman	S	U	Not tested	Not tested

*F, female; M, male; A, adult (>2 y old); S, subadult (1 to <2 y); J, juvenile (<1 y); U, unknown, CDV, canine distemper virus.
